# Complications of Blepharoplasty: Prevention and Management

**DOI:** 10.1155/2012/252368

**Published:** 2012-05-08

**Authors:** James Oestreicher, Sonul Mehta

**Affiliations:** Division of Orbital, Ophthalmic Plastic and Reconstructive Surgery, Department of Ophthalmology and Vision Sciences, University of Toronto, Toronto, ON, Canada M5S 3A5

## Abstract

Blepharoplasty is an operation to modify the contour and configuration of the eyelids in order to restore a more youthful appearance. The surgery involves removing redundant skin, fat, and muscle. In addition, supporting structures such as canthal tendons are tightened. Other conditions such as ptosis, brow ptosis, entropion, ectropion, or eyelid retraction may also need to be corrected at the time a blepharoplasty is performed to ensure the best functional and aesthetic result. Due to the complexity and intricate nature of eyelid anatomy, complications do exist. In addition to a thorough pre operative assessment and meticulous surgical planning, understanding the etiology of complications is key to prevention. Finally, management of complications is just as important as surgical technique.

## 1. Preoperative Assessment

In the initial assessment, patients are encouraged to voice their desires and concerns regarding the aesthetic appearance and functional features of their eyelids. Reassuring the patient that privacy will be maintained helps facilitate the patient's ability to articulate his or her desired outcome. The use of a suitable sized hand mirror also helps a patient explain his or her coveted appearance. If the patient continues to have difficulty describing or demonstrating what he or she desires changed, and into what, it obligates the surgeon to promote discussion or present alternatives until clear agreement occurs—otherwise, surgery should not be done.

It is important to elicit particular concerns of each individual patient, and also for the surgeon to identify unrealistic expectations. Patients' concerns can vary immensely, ranging from a particular dislike of lateral hooding, a “staring” or “overdone” look (very common), a sunken look (a common concern in younger patients), to a fear of blindness to concerns about the length of the recovery period and intra- and perioperative pain. Unrealistic expectations include those patients who desire no upper lid fold at all, operated patients (who already look over corrected) desiring further “improvement”, patients who plan to return to their high demand occupation the day after surgery or those who book travel within the first week of surgery. Patients who view cosmetic surgery as a commodity rather than a medical procedure with attendant risks should not be operated on. In the initial consultation, it is important for the surgeon to identify which unrealistic patients can be educated and operated on with confidence, and which ones cannot [1, 2]. 

Once patient's concerns are identified, the surgeon should inquire about cardiac and thyroid disease, hypertension, diabetes, bleeding diathesis, and keloid scar formation. Allergies and a list of medications should be noted. Patients taking aspirin, anticoagulants, nonsteroidal anti-inflammatory agents, vitamin E, gingko, and other herbal medications should stop them, if possible, up to 3 weeks preoperatively.

On examination of the patient, the surgeon must look for ophthalmic and periocular disease by history and a full-eye examination. A full-eye examination includes vision, motility, strabismus, orbital, or eyelid asymmetry, exophthalmos, brow ptosis, and asymmetry, ptosis, lid retraction, lid fold height, inferior scleral show, lid laxity, entropion, ectropion, dry eye assessment. Important measurements to evaluate include palpebral fissure, marginal reflex distance, amount of lagophthalmos, and lid crease height. A slit lamp examination and Schirmer's test are necessary in this author's view.

## 2. Surgical Planning

When planning to perform an upper lid blepharoplasty, determining the amount of excess skin in the upper lids, the amount of excess or prolapsed fat, the position of the lacrimal glands, and the extent of lateral hooding and medial bulging are important.

When preparing for lower lid blepharoplasty, important features to note are the amount of excess skin and the presence of fine rhytids (wrinkles), prolapsed fat (quantity and location), malar bags or festoons, lid laxity, scleral show and pigmentary characteristics. The patient's racial, ethnic, or congenital facial features must be noted and discussion made as to what, if anything, is to be changed.

Old photographs are useful to determine the patient's youthful upper eyelid fold configuration. It must be understood that old photographs do not represent a guarantee or even a goal, but rather act as a guidepost. Many people never had a full “wide open” upper lid and appeared “heavy-lidded” in younger years and their lid crease height is at 7 mm, not 10 mm. Usually, it is a mistake to try and change their upper eyelid nature too drastically, unless this desire and postoperative appearance is made abundantly clear.

Surgical planning involves deciding whether upper or lower eyelids, or both will be operated on. It also includes deciding which technique to perform (steel blade versus CO_2_ laser, transconjunctival versus external approach to lower blepharoplasty). Any adjunctive procedures to be performed should also be determined. Adjunctive procedures include brow ptosis repair (internal trans-blepharoplasty, direct, coronal, or endoscopic), ptosis repair, lacrimal gland suspension, eyelid lengthening, and lower eyelid tightening or lateral canthopexy. Lower eyelid skin excision or laser resurfacing (or neither) is another key decision.

The authors favor CO_2_ laser blepharoplasty with a trans-conjunctival lower lid approach. CO_2_ skin resurfacing is useful to address skin redundancy and festoons (in patients with appropriate skin types).

## 3. Complications

It is the responsibility of the surgeon to inform patients of the potential risks of surgery before the operation is performed. As the surgeon, it is important to be aware of the potential complications of surgery. Complications of blepharoplasty can be minor or serious. The perceived gravity of a given complication may differ between the patient and the surgeon [1, 3]. Establishing trust and communication is essential to a doctor-patient relationship, perhaps even more important in a completely elective, aesthetic procedure with high expectations and standards. Postoperatively, the management of patients' concerns can range from reassurance to surgical intervention, depending on the concern.

### 3.1. Superficial Ecchymosis and Hematoma

Bruising will be experienced by every blepharoplasty patient, so it is not really a complication so much as an expected side effect. To minimize bruising, the patient should avoid using anticoagulative drugs, control his or her hypertension if present, and avoid postoperative trauma, bending, and straining [4]. The use of the CO_2_ laser and maintaining a dry surgical field with bipolar cautery or by defocusing the CO_2_ laser will minimize the occurrence of postoperative ecchymosis. Excessive bruising can lead to a prolonged recovery, infection, cicatrisation, and skin pigmentation.

Postoperatively, the patient can aid recovery with a few simple interventions—ice water compresses and head elevation. Ice water compresses should be utilized continuously for 3 days (except when eating or sleeping). Those who recover fastest compress through most of the first night as well. Ice packs or frozen masks are too heavy, which may damage the eyelid tissues or dehisce wounds. Patients should rest with their head up at least 45 to 60 degrees. Preoperative and postoperative oral arnica (a herbal healing agent) has been claimed anecdotally to help when given in normal doses.

### 3.2. Wound Dehiscence

Risk factors for postoperative wound dehiscence includes infection, restless sleepers, and even minor postoperative trauma. Minimizing wound dehiscence involves appropriate suture choice and suture placement. For an upper lid blepharoplasty, skin sutures with 6-0 prolene imbricating levator or pretarsal tissue is preferred. Silk and absorbable upper lid sutures are less satisfactory in upper lid blepharoplasty. Absorbable upper lid sutures either in the skin or buried, have a risk of tissue reaction or dehiscence. Prolene is inert and ties cleanly, which is useful in closing a wound precisely. CO_2_ laser incisions need 7 days to heal, so sutures are removed on day 7 or 8. A running prolene suture, with several interrupted reinforcements is useful. Patient discomfort from suture removal is minimized by using Jeweller's forceps and sharp Vannas scissors.

The conjunctival incision made in a transconjunctival lower lid blepharoplasty never requires sutures. This is because they cause more harm than good. It is often necessary to tighten the lower eyelid at the time of blepharoplasty. Depending on the amount of laxity, a full lateral tarsal strip procedure or a lateral canthal tendon plication can be done. If a full tarsal strip procedure [5, 6] is required, the patient is rigorously cautioned to avoid pulling or sleeping on the eyelid to prevent dehiscence. Slight dehiscence can be treated with topical and oral antibiotics, but a complete dehiscence needs prompt debridement and repair to avoid lower lid retraction and scarring. Milder eyelid laxity is treated by a form of lateral canthal tendon plication at the time of lower lid blepharoplasty, and dehiscence here is less common and of milder extent, and hence can usually be managed supportively [7].

### 3.3. Scar Abnormalities

**Figure 1:**
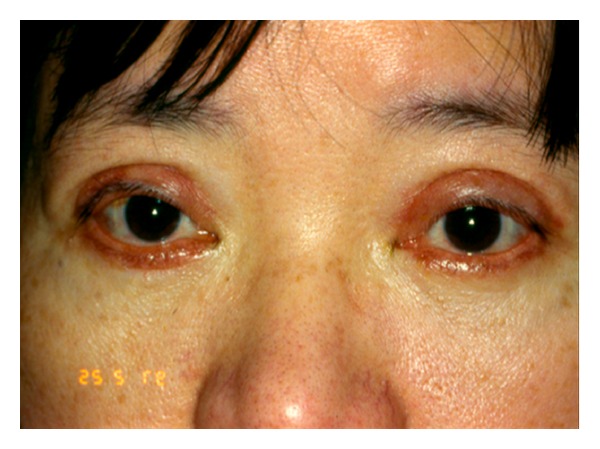
Scar Hypertrophy and dyspigmentation after transcutaneous blepharoplasty incisions done elsewhere with CO_2_ laser in an oriental patient.

Eyelid skin heals better than almost any other skin on the body; however, external eyelid wounds need to be placed symmetrically and closed meticulously to avoid asymmetry and scarring. Occasionally, incision lines may look hypertrophied, particularly in keloid-forming patients. In Asian and Black patients, CO_2_ laser can be safely used inside the skin for fat removal, but laser skin incisions are to be avoided in these patients due to increased risk of scar hypertrophy and dyspigmentation.  Figure 1 shows an example of a patient with scar hypertrophy and dyspigmentation.

If the incision line is a slightly thick and red at 4 weeks, then time, massage, and vitamin E cream is useful. Very rarely topical or injected steroids can be used, as true keloids of the eyelid skin are rare.

Occasionally instead of scar hypertrophy, epithelial inclusion cysts occur. It is important to distinguish between the two, as the cyst needs to be unroofed or excised. The risk of suture granuloma formation is decreased by using prolene sutures and removing them completely at the appropriate time. Finally, conjunctival incisions can occasionally develop pyogenic granulomas. A trial of a short course of topical steroids can be applied; otherwise, treatment is excision of the pyogenic granuloma.

### 3.4. Upper Eyelid Overcorrection

Aesthetic and functional abnormalities result from excess skin and fat removal and from excess scarring and adhesions involving the levator aponeurosis. Risk factors for overcorrection include previous eyelid trauma, dermatological conditions leading to tight skin, and Graves' disease. Measurement and precision are key to avoiding overcorrection. Generally, the surgeon must leave 10 mm of skin under the brows above the upper lid crease incision in order to avoid lagophthalmos, and more if the lid crease height is less than 10 mm from the lid margin. Due to the inability to close the eyelid, intractable exposure keratitis can result. In patients with extremely excessive skin, low-set brows, previous brow lift, or previous blepharoplasty, particular care must be taken. More effect (in terms of lifting skin off the eyelashes) for less skin excision can be achieved by creating a higher lid crease during the blepharoplasty.

**Figure 2:**
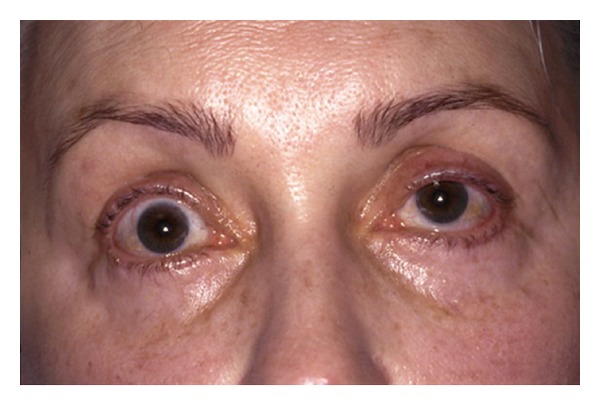
Upper lid retraction after upper lid blepharoplasty.

Excessive trauma to the levator muscle, levator aponeurosis, and pre-aponeurotic fat pad can result in upper lid retraction, scleral show, and lagophthalmos.  Figure 2 shows an example of upper lid retraction secondary to upper lid overcorrection. Scleral show can occur with excess laser energy deposition when the fat is removed. To avoid this, use a Q-tip backstop immediately behind the fat incision made by the CO_2_ laser. Also, avoid excess cautery to the levator.

Pure skin lack can be remedied by a full thickness skin graft. If the surgeon thought to preserve the excised skin in moist gauze, this can be utilized up to one week postoperatively. Retroauricular skin is often available and is a good substitute for eyelid skin. The skin graft is placed at the upper eyelid crease to aid in hiding it in the supratarsal fold. However, it will always be less cosmetic than a primary blepharoplasty done conservatively, and it may take up to one year to blend in.

If deeper scarring requires release, it should be done at the time of skin graft placement. In addition, placement of an upper lid traction suture is important or the skin graft will be ineffective [7–9]. Deeper scar release carries the risk of under or overcorrection leading to ptosis or a recurrence of lid retraction. Proper repair is an art in itself. Multiple repairs may be required for the optimum result to be achieved. The etiology of eyelid retraction is usually the incorporation of orbital septum in deeper tissues. Therefore, it is critical to release the septum from these deeper tissues. Secondary upper lid lengthening can also be done posteriorly if adequate skin grafting has already been carried out, thereby avoiding another skin incision. Another useful technique is to leave the traction suture in beyond one week. By asking the patient to pull against the levator with the traction suture will help modulate the eyelid height and achieve a more desired height. Upper eyelid spacer grafts such as sclera or tarsus are best avoided, as they are unnecessary and can be unsightly and palpable to the patient.

**Figure 3:**
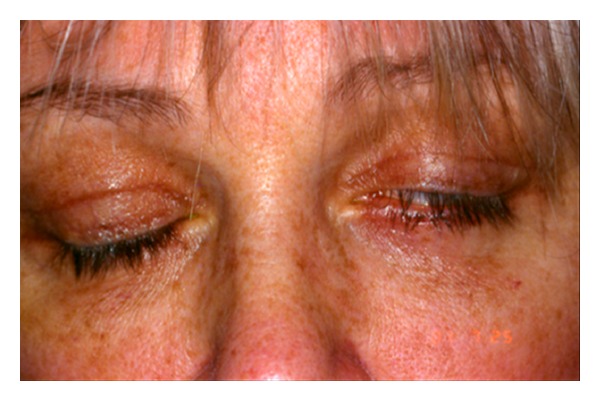
Lagophthalmos secondary to upper lid overcorrection.

Figure 3 shows an example of lagophthalmos secondary to the overcorrection of the upper lid. Because of the complexities in modifying the overcorrected upper lid, a more mild degree of symptomatic lagophthalmos can be addressed via lower lid elevation with lower lid posterior lamellar grafting, as detailed in the next section. This can improve lagophthalmos without visible external incisions or the risk of induced ptosis or unsightly skin grafts when used. The amount of lagophthalmos must be such that lower lid elevation would eliminate it. On average, this amount is between 1 to 2 mm. Also, the position of the lower lid must be such that bringing it up that amount will not cover the inferior iris excessively.

Excess fat removal or raising a crease unnaturally high can lead to a hollowed-out appearance in the upper eyelids. Even a moderate amount can be upsetting to the patient who has always been heavy lidded. Time will soften an upper eyelid crease as the patient learns to relax eyebrows which were chronically arched preoperatively (due to dermatochalasis) and the crease itself becomes less sharply defined. Filling in the hollowed areas can be problematic. Fat pearls, fat injections, dermis fat grafts, and alloplastic injections can be tried. The risks are significant and include brief effect, scarring and tissue irregularities, uneven contours, and ptosis and lid retraction. Blindness and embolic stroke can occur with accidental intravenous or intra-arterial injection of these materials, particularly near the supraorbital vessels [10, 11].

### 3.5. Lower Eyelid Overcorrection and Retraction

**Figure 4:**
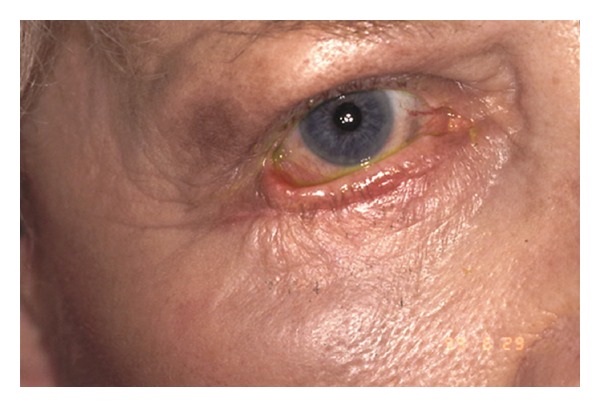
Lower eyelid of this patient shows cicatricial ectropion with middle lamellar scarring causing lid retraction as well after blepharoplasty elsewhere. The patient has severe symptomatic lagophthalmos as well as an unsightly appearance.

**Figure 5:**
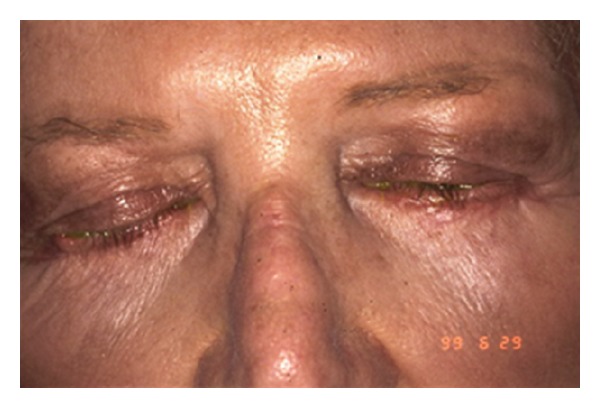
Significant lagophthalmos illustrated. The patient had symptomatic exposure keratitis despite copious lubrication and taping the eyelids closed at night.

**Figure 6:**
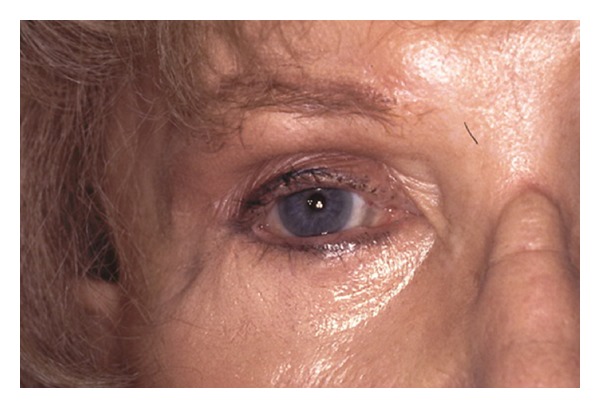
Lower eyelid of the same patient shown in Figures 4 and 5 after re-draping of the lower eyelid skin (no skin graft required), as well as lower eyelid elevation and scar release with posterior hard palate mucosal graft. There is essentially no remaining ectropion or retraction, and her lagophthalmos is also gone.

**Figure 7:**
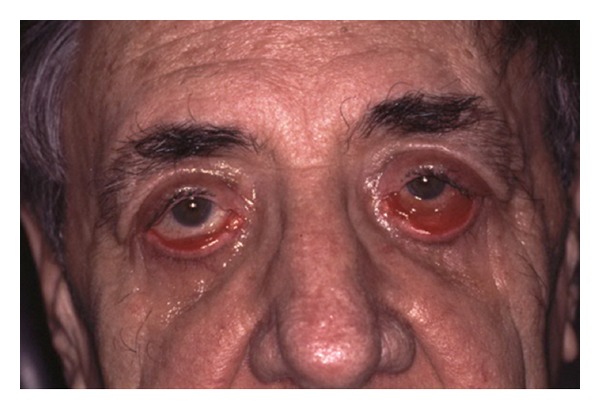
Severe lower eyelid ectropion and retraction in a patient who underwent blepharoplasty elsewhere followed by several reparative attempts by the same surgeon. The patient was given topical steroids by his original surgeon, resulting in untreated intraocular pressure of 45 OU. He had severe chemosis and discomfort due to significant lagophthalmos.

**Figure 8:**
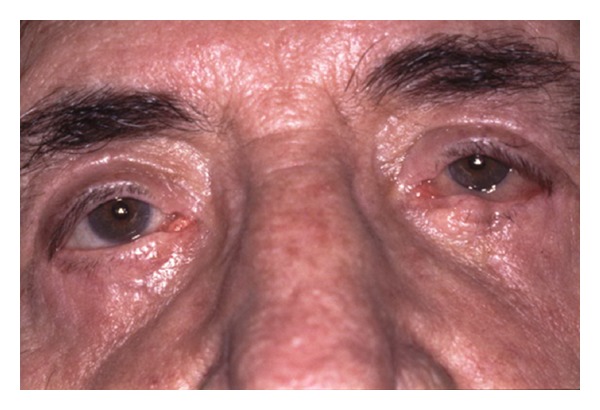
Postoperative view of patient in Figure 7 after lower lid elevation, scar release, posterior lamellar hard palate mucosal grafting, and skin graft on the left (more severe) side. The eyelids were operated on separately due to the need to patch and put them on traction for a period of time after surgery. Intraocular pressure is back to normal.

Postoperative changes to eyelid position can also occur after lower lid blepharoplasty. Abnormalities of lower eyelid position include lower lid retraction with scleral show, rounding of the lower eyelid contour, rounding of the lateral canthal angle, and ectropion. These can result from skin shortage, middle-lamellar (orbital septum) scarring, and posterior lamellar (retractors and conjunctiva) cicatrisation as seen in Figures 4, 5, 6, 7, and 8. The horizontal laxity of the tarsoligamentous sling of the lower eyelid is often overlooked at the time of surgery, which allows the other abnormalities to manifest themselves after surgery [12, 13].

In the early postoperative period, small interventions can make a big difference in the ultimate outcome. Treatment of conjunctival chemosis can alleviate downward pressure on the lower eyelid. Elimination of topical allergy, and occasionally short-term topical steroid use are helpful. The patient can be instructed in upward massage to keep infection and scarring minimized and alleviate retraction. If early cicatrix formation is detected, local nondepot steroid injection can occasionally eliminate the need for more involved surgery. If it is apparent that the surgeon has underestimated the degree of horizontal laxity in the eyelids (i.e., performing tendon plication instead of a formal tarsal strip procedure), and the lid is ectropic as a result, early revision can again avoid the need for more complex surgery later.

Graded eyelid horizontal tightening is utilized in all but the youngest patients. Transconjunctival fat resection alone should be considered in younger patients who may have very little excess skin and whose skin may be resilient enough to tighten itself spontaneously postoperatively. Laser resurfacing is utilized where skin shrinkage and rhytid reduction are desired. The subciliary skin muscle flap approach to the fat pads is avoided if at all possible. In patients (especially males) with prominent skin and orbicularis excess who are not laser candidates, fat is still removed transconjunctivally, the eyelid is tightened horizontally and a conservative skin muscle pinch excision is utilized. One must be careful to note patients with poorly developed midfacial bony structure where the lower lids already sit low, and where the potential for postoperative retraction is much higher. Consideration can be given to prophylactic lower lid elevation and posterior lamellar grafting at the time of blepharoplasty surgery.

In late cases, the relative contribution of lid laxity, skin shortage, and middle lamellar scarring is assessed by the “three finger test”. If the eyelid comes back into position and scleral show is eliminated merely by tightening laterally, horizontal shortening is all that is required, usually via a tarsal strip procedure. (Remember there is an increased rate of dehiscence of the periosteal attachment in these circumstances.) If a second finger is required in the central eyelid pushing upward, usually a posterior-lamellar graft is required. If skin shortage is evident however, full-thickness skin grafting may be needed. In equivocal cases, a posterior lamellar graft can be tried first, and the patient warned that a following procedure with a skin graft may be necessary. Hard palate mucosa is commonly utilized for the graft [14–19]. A free tarsoconjunctival graft can alternatively be used [20–23]. If a third finger is required to recruit skin by pushing the mid face up, skin grafting or possible mid face lifting may be necessary. A partial improvement may be achieved with a posterior lamellar graft and horizontal tightening alone.

The technique of tarsal strip repair has been well described elsewhere. The skin and orbicularis, lid margin, conjunctiva, and lower lid retractors are removed from the excess eyelid laterally, creating a lateral tarsal strip which is then anchored to Whitnall's tubercle inside the lateral orbital rim. The lateral canthal angle is reformed to an acute configuration [24–26].

Posterior eyelid elevation is achieved by careful dissection at the level of the bottom of tarsal plate through conjunctiva, lower lid retractors, and orbital septum, and these are recessed downwards off the overlying orbicularis muscle. Visualized and palpated scar is released aggressively in the postblepharoplasty retraction circumstance, so the lid is freed from attachments to the inferior orbital rim. A posterior lamellar graft is then placed between the cut lower edge of tarsal plate and the recessed cut conjunctival edge. Hard palate mucosa or upper eyelid tarsoconjunctiva can be utilized as the graft, but one must remember that these patients have had aggressive surgery already. It is, therefore, often wise to avoid further manipulation of the upper lid by taking a donor graft from it. The lower lid is then tightened if lax or given an upward vector with a minimal Elschnig tarsorrhaphy if not lax. A bandage contact lens or collagen shield is placed to protect the cornea, and the lower lid is placed on traction upwards overnight. These techniques are similar to those utilized to treat the eyelid retraction of thyroid eye disease [27].

Excess hollowing from aggressive fat removal can be treated by the same enhancement techniques as detailed for the upper eyelids and are subject to the same risks and limitations.

When skin shortage dictates skin graft placement, the technique is similar to that for other forms of cicatricial ectropion. The previous scar is opened up, internal adhesions are widely released (and perfect hemostasis obtained). The lid is placed on upward traction to facilitate this process, and an appropriately sized full-thickness graft is contoured to fit the defect after the eyelid is tightened horizontally. The lid should be kept on upward traction 1 to 7 days with a frost suture to the lateral eyebrow [28, 29]. Midfacial lifting is beyond the scope of this monograph [30, 31].

### 3.6. Asymmetry

Meticulous preoperative planning, including precise measurements and noting any asymmetry in facial features, should be a routine for every surgeon. Not only the surgeon but also the patient should be aware of preoperative asymmetry and the potential for minor “touch up” operations. These should usually be delayed for 3 months or more if possible after the primary procedure to avoid surgical “tail chasing.” Allowance for asymmetry not to be corrected (such as minor brow height differences) needs to be made.

The most common result which will be noted by the patient is lid crease asymmetry. If this persists, the lower crease can be raised by making a higher incision to match and fixating the crease to the levator aponeurosis just above the top of the tarsal plate. It is difficult to lower a crease which is too high. The risk is failure, with reemphasis, doubling, or other scarring of the existing low crease. If essential, a lower incision is made and fat is teased forward between the skin and levator to prevent readhesion of these structures.

Another outcome noted by patients is asymmetry of lateral hooding reduction. Careful preoperative marking will minimize the incidence of this result and of course many minor degrees of asymmetry will disappear with time. If persistent, a superolateral skin excision with crease reformation will raise the persistently hooded side. It is important to tailor the incision upwards at the lateral extent or the hooding will persist.

**Figure 9:**
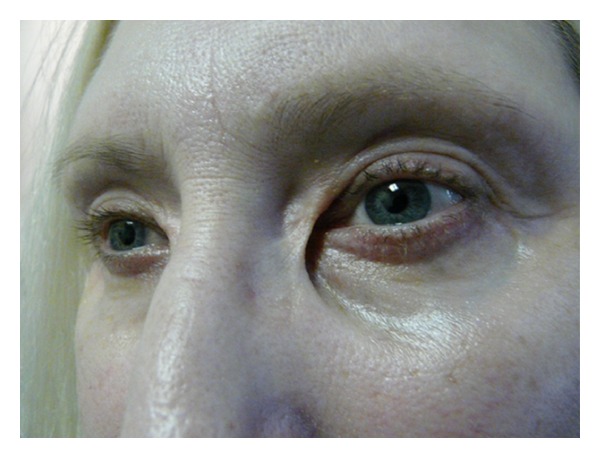
Medial canthal webbing seen after upper lid blepharoplasy done by a dermatologist.

Medial canthal webbing occurs when incisions are carried too medially as seen in Figure 9. The skin then bridges the superomedial hollow of the upper lid in a straight line. Early recognition and aggressive massage will eliminate the majority of cases. Persistent cases are treated by a V- to-Y plasty procedure.

### 3.7. Ptosis

Ptosis of varying degree is common for patients to experience the day after upper lid blepharoplasty. The experienced surgeon who is certain that the levator muscle and aponeurosis was identified and preserved during surgery will not be alarmed. Postoperative eyelid edema and levator edema are common and are temporary causes of ptosis. Remember that the levator aponeurosis is the stage on which the fat removal of upper blepharoplasty is played; and it is natural for early postoperative dysfunction to occasionally be seen.

However, certain caution should be taken to avoid and manage postoperative ptosis. The surgeon must know his or her patient's anatomy and distinguish septum from levator. Septum must be opened if fat is to be removed, but not the levator. The two fuse low in the upper eyelid, so the inexperienced surgeon is well advised to open the septum high up where there is a good barrier of preaponeurotic fat underneath to protect the levator. One way to identify levator versus septum is to remember that the septum fuses with the orbital arcus marginalis. If the orbital septum is pulled, the surgeon can feel it tighten when a finger is placed under the brow. Similarly, if the patient is asked to look up, the orbital septum will not move when grasped but the levator will. Remember also that when the preaponeurotic fat is grasped and the septal attachments divided, it is possible to pull the superficial levator aponeurosis up with it. Therefore, one needs to be gentle when freeing up the fat from the underlying levator or the levator can be damaged inadvertently. Similarly, when using the CO_2_ laser to cut fat lobules free, one needs a “back stop” (usually a Q-tip) to absorb the transmitted laser energy and avoid damage to the structures that lie beneath (levator, Muller's muscle, conjunctiva and globe). The same principle applies in lower lid fat removal to protect the inferior oblique.

If a definite levator laceration is observed, it should be repaired if it is causing ptosis. It may be necessary to lighten the patient's sedation to gain an accurate assessment of lid height, and sitting them upright is also useful. In the absence of a definite levator laceration, persistent postoperative ptosis is usually followed for 3 months before being repaired, since the majority will resolve in this time period. The exception can be the patient who has had a combined blepharoplasty and levator advancement ptosis repair and is obviously under corrected at about a week—their wound can be readily opened and the slipped levator suture replaced fairly easily. However, another approach to management to postoperative ptosis is to wait the 3 months and then perform a posterior Fasanella-Servat procedure. This fast and predictable approach avoids opening the anterior wound and also avoids overcorrection and scar abnormalities.

### 3.8. Epiphora and Ocular Discomfort

Blink dysfunction is common postblepharoplasty because of postoperative swelling of the eyelid tissues. This interferes with the tear pump mechanism. Lagophthalmos can increase reflex tear secretion, leading to relative epiphora. The swelling can also cause the puncta to turn inwards or evert by swelling or tissue contraction caused by incision lines or laser resurfacing, which also causes epiphora. Similarly, conjunctival chemosis caused by a transconjunctival incision and by drying related to lagophthalmos can cover the puncta, again leading to epiphora. Lubrication, cool compresses, and observation are essential to resolution. Similarly, corneal epithelial breakdown can result in transient pain, foreign body sensation and tearing. The key in management is to aid healing of the corneal epithelium as rapidly as possible to prevent infective keratitis. Ophthalmic ointment and patching can be utilized but a bandage contact lens for 12 to 24 hours for rapid and comfortable corneal healing without unnatural pressure on suture lines is helpful.

Epiphora from damage to the lacrimal outflow system can occur if the incision line is carried too medially and too close to the horizontal midline. The punctum is a useful landmark for the upper lid and lower lid incision. For an upper lid blepharoplasty, ending the incision just lateral to the punctum avoids medial canthal webbing as well as lacrimal system injury. Incisions should be at least 4 to 5 mm above the punctum to avoid the canaliculus. Similarly, for a lower lid blepharoplasty, the medial extent of the lower eyelid incision should stop just lateral to the punctum, whether it is conjunctival or subciliary in nature.

True canalicular injury may require late repair if epiphora results. Many older patients do not have tearing with one obstructed canaliculus due to decreased tear production. If the obstruction is more distal than 8 mm from the punctum (unlikely in blepharoplasty surgery), a canaliculo-dacryocystorhinostomy may reconstruct the system. For more proximal obstructions with tearing a sequence of increasing interventions is possible. One starts with a three snip on the punctum of the unobstructed canaliculus, followed by a DCR (to enhance flow through the unobstructed canaliculus), followed by a DCR with Jones tube in refractory cases.

### 3.9. Diplopia

Fortunately, diplopia after blepharoplasty is extremely rare but is still a known complication. The commonest form is caused when local anaesthetic is supplemented intraoperatively by direct fat injection once the conjunctiva (lower lid) or skin (upper lid) is open. This is due to more rapid and wider diffusion of the local anaesthetic agent, affecting other structures such as cranial nerves. One should identify (and preserve) the inferior oblique and levator during surgery, to be confident they have not been injured. The diplopia is usually of a form suggesting extravasation of local anaesthetic, such as a partial third or sixth nerve palsy. If concerned, the patient can be observed until signs of improvement are noted. Despite the use of a lidocaine/marcaine mixture for local anesthetic, it is important to note that this form of diplopia is always gone by the next day.

Another mechanism is direct or indirect injury to the inferior oblique during surgery. Injury to the inferior oblique or less commonly other extraocular muscles, is rare. One of the signs of imminent damage to the muscle is excess bleeding. The surgeon needs to stop the bleeding but at the same time avoid excess cautery or other trauma to the muscle. The oblique divides the medial lower fat pad from the central lower fat pad and it should be easily identified, and thus protected. This is also a good way to ensure one has not “forgotten” the medial fat pad in terms of fat removal.

Persistent diplopia beyond the first day will often resolve with eye movement or fusion exercises, if there is no gross deficit. The assistance of your strabismus-oriented colleagues can be occasionally very helpful if the deficit persists. Lastly, there are occasional patients who develop unrelated cranial nerve palsies some weeks or months after surgery by chance alone. These are investigated and followed in the normal fashion for such conditions.

### 3.10. Ocular Injury

Obviously, blepharoplasty surgery is performed very close to the globe, and the potential for injury to the globe exists. Increased risk exists in the patient with proptosis, such as a patient with thyroid eye disease or the patient with a large or projecting glaucoma bleb. Globe injury can occur with the CO_2_ laser, with a steel scalpel, or with local anaesthetic injection.

Laser eye protectors are essential if the CO_2_ laser is utilized, but there must be enough ocular lubrication present to avoid a corneal abrasion when they are inserted or removed. The laser must always be directed away from the globe even through eye shields are in place. Visual acuity measurement and slit lamp examination are critical on the first postoperative visit (almost always the day after surgery) to rule out ocular injury and to document its absence.

**Figure 10:**
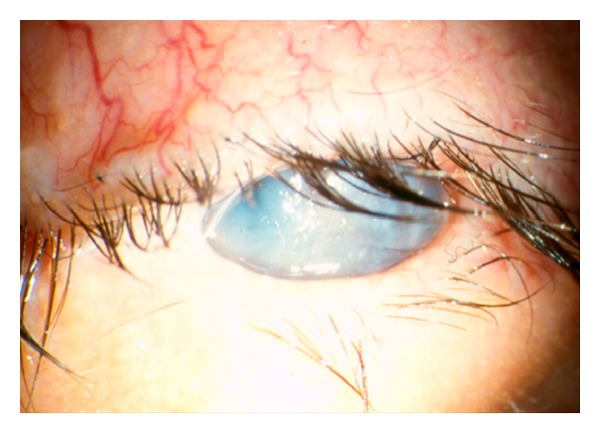
Severe corneal scarring secondary to severe lagophthalmos after blepharoplasty done in a patient with Thyroid Eye Disease.

Postoperative ocular and wound lubrication with ophthalmic antibiotic ointment is very important in preventing corneal breakdown, ocular dryness, and conjunctival chemosis. This is because most patients will initially experience small amounts of lagophthalmos from ongoing local anaesthetic effect on the orbicularis, swelling, and stiffness of the eyelids.  Figure 10 shows corneal scarring due to severe lagophthalmos.

A vicious cycle can develop wherein the chemotic conjunctiva dries out because it is swollen and then swells because it is dry. This can also lead to corneal dellen formation, or a dry cornea can break down *de novo*. Patients should plan to not drive for a week, due to the blurriness caused by the ointment use.

In the setting of blepharoplasty surgery noninfected corneal abrasions are best treated with a bandage contact lens. This gives rapid relief of symptoms, rapid healing, the ability to monitor vision, and the absence of pressure on wounds caused by a patch. A contact lens does require a daily or near daily visit until the abrasion is healed and the lens is removed.

Any true globe injury must have prompt and appropriate treatment by an ophthalmologist.

### 3.11. Orbital Hemorrhage with Vision Loss

Recognizing that orbital haemorrhage with vision loss is a possible although rare complication from blepharoplasty surgery is important. The incidence is estimated to be 1 in 2,000 to 1 in 25,000 [32]. Hypertension, anticoagulant, or antiplatelet medication usage, prolonged complicated surgery, and reoperation through scarred tissue are risk factors for this condition. Retrobulbar hemorrhage is a form of compartment syndrome, with the orbit bounded by four bony walls and the orbital septum acting as the compartment. With an acute hemorrhage, intraorbital pressure rises abruptly, and the blood supply to the optic nerve is compromised. Any concomitant rise in intraocular pressure is secondary and treating it will not affect outcome.

Recognition is key, as is a rapid response. Proptosis, decreased motility, and increased orbital tension, and associated bleeding are the clinical signs to appreciate. The patient will also have asymmetrical pain and decreased vision. If suspicious that an orbital hemorrhage has occurred, laser eye protectors (metallic scleral contact lenses) block vision and must be removed to assess the visual acuity. Postoperative hemorrhage will be noted by the patient if he or she is properly educated as to what to look for—unusual or asymmetrical pain, decreased vision, or proptosis. Patients must be taught to check their vision one eye at a time. An effective emergency contact arrangement needs to be in place so prompt assessment and intervention can be carried out [33].

Rapid treatment is critical. Control of obvious bleeding points, if present is important. However, rapid release of orbital pressure by opening the wound, lateral canthotomy and inferior and/or superior cantholysis is critical. The surgeon should spread bluntly posteriorly into the orbit down the lateral wall and through the wounds to access deep hematomas and release them. If done in the plane of the lateral wall and in the plane of the levator aponeurosis and inferior rectus (i.e., parallel to these structures) in a blunt fashion the risk of significant damage to orbital structures is low. In the face of frank orbital hemorrhage with proptosis, a frozen globe, and vision loss, bold measures are called for. Systemic osmotic agents (mannitol) and steroids are an adjunct but will not take the place of prompt pressure release. It is rare that true bony decompression either at bedside through the inferomedial floor or more fully in the operating room is required. Antiglaucoma medications or anterior chamber drainage are treatments aimed at central retinal artery occlusion, not orbital hemorrhage. CT scanning the orbits is important, but only after treatment has been carried out. Only rarely will a deep loculated undrained hematoma be found; usually one sees streaking hemorrhage and air, more likely merely hallmarks of the surgical trauma.

Unfortunately, treatment beyond 1 to 6 hours of total or near-total vision loss is unlikely to be effective. Up to 24 hours, cantholysis and pressure release (if the orbit is still tense) and steroid treatment can be utilized. Beyond this time period, one may be over treating the patient and exposing them to additional complications with very little prospect of improvement. After 24 hours of “spinal-trauma” dose level of steroids (solumedrol 30 mg/kg bolus over 15 minutes followed by 5.4 mg/kg per hour) without response, one can discontinue the drug, possibly after repeat imaging.

Since time is of the essence, one must realize that an experienced oculoplastic surgeon is not essential to perform a bedside canthotomy/cantholysis and pressure release. All ophthalmologists should feel comfortable treating orbital hemorrhage with canthotomy and cantholysis.

Posttreatment admission to hospital is recommended, with close visual acuity monitoring, head elevation, ice water compresses, intravenous steroids until 24 hours of stable vision have been noted, as well as imaging with CT scanning. Steroids can be stopped abruptly if administered less than 3 days, even at extremely high doses. Topical and systemic antibiotics are utilized due to the open wounds, and their repair is planned electively in 1 to 2 weeks if they do not close on their own. Improved vision needs to be monitored by hospital staff or by the patient for stability for 1 to 3 days after treatment is stopped.

### 3.12. Pigmentary Abnormalities and CO_2_ Laser Resurfacing

Many patients present for correction of “dark circles under the eyes.” “Dark circles” are caused by 3 factors: shadowing caused by fat bulging above the dark area, the blood supply of the fat showing through the thin eyelid skin, and thirdly, actual pigment in the epidermis and dermis. Fat removal will help the first two causes, and laser skin resurfacing can aid the third if the pigment is relatively superficial. The patient must be a resurfacing candidate to consider this treatment modality (Fitzpatrick skin type, I, II, or III), and the risks of hypopigmentation and hyperpigmentation stressed. If pigment is present without fat herniation, treatment with skin bleaching agents can be tried first. In darker-skinned patients at risk for reactive posttreatment hyperpigmentation, pre and posttreatment with topical Retin-A and bleaching creams can be utilized. Various compositions of bleaching creams have been published, containing combinations of hydroquinone, glycolic acid, kojic acid, retinoic acid, and hydrocortisone.

**Figure 11:**
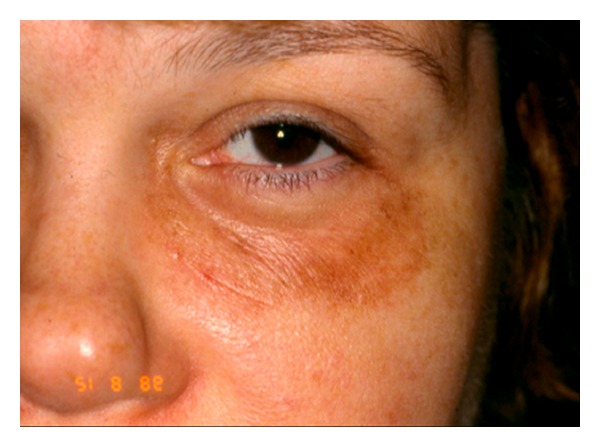
Hyperpigmentation following CO_2_ laser resurfacing.

Nonlaser-induced postoperative hyperpigmentation can result from hematoma formation and excess sun exposure. Laser resurfacing itself carries a risk of hypopigmentation (very rare in the eyelid skin) and hyperpigmentation.  Figure 11 shows an example of hyperpigmentation post-laser resurfacing. Patients with vitiligo may have an increased risk of hypopigmentation. A test spot can be offered the patient although a good result with the test spot is not a guarantee of subsequent good results. There is no consistently effective treatment of hypopigmentation. Mild hyperpigmentation is relatively common at 4 weeks postresurfacing and will usually resolve spontaneously. If noted, however, it should be treated with bleaching creams. If persistent, intense pulse light is a useful adjuvant treatment.

Postlaser-resurfacing erythema is universal and expected. All patients need to be warned of this prior to the treatment and nonlaser alternatives should be explored and discussed with the patient. Laser resurfacing in appropriate patients combined with transconjunctival blepharoplasty and appropriate lid tightening gives a far superior result to conventional exterior blepharoplasty, in terms of scar avoidance, avoidance of eyelid retraction, and a more natural and complete resolution of skin redundancy and rhytids.

The erythema lasts an average of 3 months in women but can be covered readily with make up after 8 or 9 days. Men seem to have ruddier skin, and the erythema last 60% as long on average. Pronounced or prolonged erythema is relatively uncommon and can be treated with topical 1% hydrocortisone cream or intense pulsed light treatments. It is virtually unheard of for this to fail to resolve.

### 3.13. Asian Blepharoplasty

Understanding the differences in anatomy in the occidental and oriental eyelid is essential when performing blepharoplasty surgery in this population. In the Asian upper eyelid, there is a lower fusion point between the orbital septum and the levator aponeurosis, which allows orbital fat to descend further down in addition to the increased fat in the preseptal fibroadipose layer.

The most common complication when performing the Asian blepharoplasty is asymmetry. Therefore, careful incision planning and meticulous surgery will minimize this problem. Avoid placing the crease too high to prevent the appearance of over-westernization.

In younger patients, crease formation by skin fixation to the anterior tarsal plate rather than the levator aponeurosis avoids ectropion of the upper eyelid margin and superior migration of the fold. Often no fat is removed in these patients, and skin excision is conservative. This skin incision height is often quit low, 3 to 5 mm depending on the preoperative consultation measurements. In older patients with excess upper lid fat, the septum needs to be formally opened to remove preaponeurotic fat. The skin incision should still be kept low, perhaps at 5 to 6 mm at the most. Crease formation should not be high on the levator (if above tarsal plate at all) to avoid a distorted “westernized” look, asymmetry, and ptosis.

For lower eyelid blepharoplasty in Asians, transconjunctival fat removal yields far superior results to an external approach [34]. 

## 4. Summary

Blepharoplasty is a widely practiced successful operation. However, because of the complex structure and function of the eyelids, the potential for complications does exist. With appropriate case selection, thorough discussion with surgical candidates, and careful surgical technique, most of these can be avoided. Effective techniques do exist to treat most, if not all, complications, which may arise.
